# A Systematic Review and Meta-Analysis of the Relationship Between Social Dominance Status and Common Behavioral Phenotypes in Male Laboratory Mice

**DOI:** 10.3389/fnbeh.2020.624036

**Published:** 2021-01-20

**Authors:** Justin A. Varholick, Jeremy D. Bailoo, Ashley Jenkins, Bernhard Voelkl, Hanno Würbel

**Affiliations:** ^1^Department of Molecular Genetics and Microbiology, College of Medicine, University of Florida, Gainesville, FL, United States; ^2^Division of Animal Welfare, Veterinary Public Health Institute, Universität Bern, Bern, Switzerland; ^3^Department of Cell Biology and Biochemistry, School of Medicine, Texas Tech University Health Sciences Center, Lubbock, TX, United States; ^4^Department of Civil, Environmental, and Construction Engineering, Texas Tech University, Lubbock, TX, United States; ^5^Department of Biology, College of Liberal Arts and Sciences, University of Florida, Gainesville, FL, United States

**Keywords:** social dominance, behavior, systematic review, meta-analysis, reproducibility, preclinical, experimental design

## Abstract

**Background:** Social dominance status (e.g., dominant or subordinate) is often associated with individual differences in behavior and physiology but is largely neglected in experimental designs and statistical analysis plans in biomedical animal research. In fact, the extent to which social dominance status affects common experimental outcomes is virtually unknown. Given the pervasive use of laboratory mice and culminating evidence of issues with reproducibility, understanding the role of social dominance status on common behavioral measures used in research may be of paramount importance.

**Methods:** To determine whether social dominance status—one facet of the social environment—contributes in a systematic way to standard measures of behavior in biomedical science, we conducted a systematic review of the existing literature searching the databases of PubMed, Embase, and Web of Science. Experiments were divided into several domains of behavior: exploration, anxiety, learned helplessness, cognition, social, and sensory behavior. Meta-analyses between experiments were conducted for the open field, elevated plus-maze, and Porsolt forced swim test.

**Results:** Of the 696 publications identified, a total of 55 experiments from 20 published studies met our pre-specified criteria. Study characteristics and reported results were highly heterogeneous across studies. A systematic review and meta-analyses, where possible, with these studies revealed little evidence for systematic phenotypic differences between dominant and subordinate male mice.

**Conclusion:** This finding contradicts the notion that social dominance status impacts behavior in significant ways, although the lack of an observed relationship may be attributable to study heterogeneity concerning strain, group-size, age, housing and husbandry conditions, and dominance assessment method. Therefore, further research considering these secondary sources of variation may be necessary to determine if social dominance generally impacts treatment effects in substantive ways.

## Introduction

AROUND the mid-20th century, scientists began to document and to understand that wild or laboratory mice residing within groups could be categorized individually by their social dominance status (Uhrich, 1937; Crowcroft, 1966; Scott, 1966; Desjardins et al., 1973). In the time since, growing evidence has suggested that social dominance status is associated with variability in behavior and physiology, where dominant mice within a hierarchy have markedly different phenotypic traits than subordinate cage-mates—despite similarity in genetics and cage-context (Lathe, 2004; Freund et al., 2013; Wang et al., 2014; Williamson et al., 2016a; Lee et al., 2018; Varholick et al., 2018, 2019). Given the pervasive use of mice throughout animal research and that they are commonly housed in groups to account for their social needs (National Research Council, 2011; Bailoo et al., 2018), understanding the biological differences between dominant and subordinate cage-mates is of significant interest (Lathe, 2004). Moreover, neglecting social dominance status in experimental designs and statistical analysis plans may inadvertently lead to the masking of treatment effects and/or contribute to idiosyncratic patterns of experimental results and in turn, poor reproducibility, if social dominance status interacts with the treatment of interest (Würbel, 2002; Bailoo et al., 2014; Varholick et al., 2018, 2019; Voelkl et al., 2020).

Social dominance relationships are often determined by observing predictable patterns regarding which animal retreats (i.e., subordinate) or chases (i.e., dominant) during social interactions (Drews, 1993). These predictable patterns may then be organized in a hierarchical fashion depending on the number of animals in the group and their dominance relationships with each other. Although different organizations of relationships can be defined, it is thought that one of the greatest differences in social dominance experience within the cage and in phenotypic traits is between the most dominant and most subordinate cage-mates, especially in male mice (Bernstein, 1981; Williamson et al., 2017a; Lee et al., 2019b). This sex difference is likely because dominant and subordinate males often engage in more overt forms of agonistic behavior (e.g., chase, lunge, bite, flee) (Lee et al., 2019a) while female laboratory mice engage in more covert forms of agonistic behavior (e.g., side-push, or over-climbing) (Schuhr, 1987)—albeit males are also studied more often than females. To determine whether dominance behavior in the home-cage is linked to differences in other phenotypic traits, scientists typically measure individual social dominance status using the gold-standard of home-cage observation or with a variety of correlated assays (e.g., tube-test or urine marking assay) (Wang et al., 2014). Scientists may then make comparisons between cage-mates of different social rank on common measures of behavior used for the screening of phenotypes (e.g., open field or elevated plus-maze) (Wahlsten, 2011).

To date, a number of studies have reported significant behavioral differences between dominant and subordinate mouse cage-mates (Hilakivi et al., 1989; Hilakivi-Clarke and Lister, 1992; Ferrari et al., 1998; Vekovishcheva and Sukhotina, 2000; Bartolomucci et al., 2001, 2004; Palanza et al., 2001; Fitchett et al., 2005a, 2009; Sá-Rocha et al., 2006; Saldívar-González et al., 2007; Wang et al., 2011; Colas-Zelin et al., 2012; Horii et al., 2017; Larrieu et al., 2017; Zhou et al., 2017; Kunkel and Wang, 2018; Pallé et al., 2019; Varholick et al., 2019), but whether such differences generalize to male laboratory mice used in biomedical research remains unknown. To provide an initial evaluation of the relationship between social dominance status and behavioral phenotype, we conducted a systematic review and ran meta-analyses when sufficient data were available. We discuss these findings in relation to the heterogeneity in the methods for measuring dominance across experiments and with respect to risk of bias.

## Methods

### Search Strategy

Using pre-specified inclusion and exclusion criteria we identified all publications reporting relevant experiments (see below) by searching three electronic databases (PubMed, ISI Web of Science, and EMBASE) using the search strategy “(anxiety OR arousal OR learned helplessness OR explor^*^ OR choice OR learn^*^ OR cognition OR preference OR motor OR pain OR maze) AND (‘social status' OR ‘social rank' OR ‘social dominance' OR ‘dominance hierarchy' OR ‘social hierarchy' OR submiss^*^) AND (mouse OR mus OR mice),” with search results limited to title and abstracts. The cut-off date for our search was on September 20, 2019. Further details on the search strategy can be found in the supplement (Supplementary Text 1). This study is in accordance with PRISMA guidelines and the Systematic Review Center for Laboratory Animal Experimentation (SYRCLE) (Hooijmans et al., 2014); the checklist can be found in the Supplementary Information section.

### Inclusion and Exclusion Criteria

One investigator (JAV) retrieved and reviewed all publications. First the titles and abstracts of 30 randomly selected papers from the 696 (~4.3%) were screened to develop the key exclusion criteria. From this screening process we set the following exclusion criteria: studies were excluded if they: (i) did not include mice; (ii) used mice housed singly; (iii) did not report measures of social interactions between cage-mates; (iv) were part of a symposium/conference proceedings or review; or (v) were written in a language other than English. In the next screening step only studies were included that housed mice in static groups/pairs for 2 weeks or more, reported methods for measuring social dominance behavior, and reported behavioral tests from the following domains: anxiety, arousal, learned helplessness, exploration, preference, learning/cognition, motor, pain, or social, outlined and described in, “*Mouse Behavioral Testing: How to use mice in behavioral neuroscience*” (Wahlsten, 2011), page 40. Studies or data were excluded if: (i) dominance relationships were measured between non-cage-mates; (ii) treatments were administered in addition to behavioral phenotyping (only control groups from these studies were used); or (iii) data from the same study were published more than once (no studies met this criterion). A detailed study protocol and flow diagram of the search and exclusion process can be found in the supplementary (Supplementary Text 1 and Supplementary Figure 1).

### Risks of Bias and Quality Assessment

Assessment of risk of bias and study quality were conducted independently by two reviewers (JAV and AJ) using a modified SYRCLE Risk of Bias Tool (Hooijmans et al., 2014) with the inclusion of sample size calculation (see Supplementary Table 1 for more details) for each of the 20 studies that met the prespecified inclusion/exclusion criteria. Any disagreements were resolved by consensus—these were low (<6%).

### Data Extraction

After compiling a final list of the 20 included studies, two reviewers (JAV and AJ) independently extracted the sample sizes, means, and standard deviations for each dominant and subordinate comparison (i.e., experiment) made within each study. For example, if a study compared dominant and subordinate mice on exploration in the open field; the sample size, mean value of exploration, and standard deviation of exploration was extracted for dominant male mice and the respective values for subordinate male mice were also extracted. This was done for each metric of each experiment within a study (a total of 99 metrics, across 55 experiments, across 20 studies). When studies housed more than two mice per cage, only the most dominant and most subordinate rankings were considered—intermediate rank assigned mice were excluded. Data were either copied directly from tables, calculated from data provided by the respective corresponding author (Larrieu et al., 2017; Varholick et al., 2018, 2019) or extracted using “Web Plot Digitizer” (Rohatgi, 2018). If data were not reported because there were null effects, the unreported data with a null effect was noted but not included in the meta-analyses (specifics provided in results). Once all data were collected by the two reviewers (JAV and AJ), a mean value for each mean and each standard deviation extracted was calculated and rounded to the nearest hundredths place. These mean values calculated between the two reviewers were used in the meta-analyses and reported tables. Both reviewers agreed on all sample sizes across the experiments.

### Statistical Analyses

All meta-analyses were calculated using jamovi (Jamovi. jamovi, 2020) and the MAJOR module (Hamilton, 2018). Jamovi is a Graphical User Interface (GUI) version of R, and MAJOR is based on the commonly used R package, Metafor (Viechtbauer, 2010). Separate meta-analyses were run for behavioral tests that had been used by 5 or more studies—the open field, elevated-plus maze, and Porsolt forced swim test. Because various outcome measures were often reported across studies for the same behavioral test (e.g., time spent in open arms, total distance traveled, number of open arm entries), we used the most frequently reported measure across studies for the meta-analysis. If a study did not use the most frequently reported measure, then we used the second most frequently used measure, and so forth. For example, the most frequently used measure for the open field was total distance traveled, followed by number of crossings on a grid, then velocity (cm traveled per second). More specifics can be found in each respective results subsection and all data can be found in the supplement.

Due to the high degree of heterogeneity between studies, meta-analyses were run by fitting a random-effects model using the standardized mean difference between dominant and subordinate mice for each respective outcome measure for each study. The sample sizes for each respective dominance status group were used for calculating each standardized mean difference for the meta-analysis. Sample sizes were determined by the number of animals per each dominance status group, the number of dominants and the number of subordinates, separately (intermediate rank assigned mice were excluded). A restricted maximum-likelihood (REML) estimation was used for calculating the heterogeneity statistic Tau^2^. No moderator was used. Behavioral tests (i.e., experiments) for which fewer than 5 studies were available, were categorized within their respective domain described in, “*Mouse Behavioral Testing: How to use mice in behavioral neuroscience*” (Wahlsten, 2011), page 40. Hedge's g was then calculated for the metric with the largest effect size for each experiment. General comparisons (e.g., smaller vs. larger effect sizes) were made between studies within their domain foregoing any further statistical testing.

## Results

### Study Characteristics

By electronic search we identified 20 studies (i.e., published manuscripts) (Hilakivi et al., 1989; Hilakivi-Clarke and Lister, 1992; Ferrari et al., 1998; Vekovishcheva and Sukhotina, 2000; Bartolomucci et al., 2001, 2004; Palanza et al., 2001; Fitchett et al., 2005a, 2009; Sá-Rocha et al., 2006; Saldívar-González et al., 2007; Wang et al., 2011; Colas-Zelin et al., 2012; Horii et al., 2017; Larrieu et al., 2017; Zhou et al., 2017; Kunkel and Wang, 2018; Varholick et al., 2018, 2019; Pallé et al., 2019) divided into 55 separate experiments that met our pre-specified inclusion criteria (Methods and Supplementary Text 1). These studies varied concerning strain, supplier, group-size, and whether littermates were housed together (Table 1). Almost half of the studies used outbred mice of various strains (*n* = 9/20), one study used inbred Balb/cJ mice, while the remainder used sub-strains of the C57BL/6 mouse (*n* = 9/20). Group-sizes varied from 2 to 9 mice per cage and only two studies housed litter-mates together.

**Table 1:** Heterogeneity in strain, group-size, and littermates for studies meeting pre-specified criteria.

**Study**	**Strain**	**Supplier**	**Group-size (# mice per cage)**
Bartolomucci et al. (2001)	Swiss CD-1^∧^	Charles River, Italy	3
Bartolomucci et al. (2004)	Swiss CD-1	Charles River, Italy	3
Colas-Zelin et al. (2012)	CD-1	Harlan Sprague Dawley Inc., Indianapolis, IN	3
Ferrari et al. (1998)	Swiss-Webster	Banting & Kingman, UK	5 to 6
Fitchett et al. (2005a)	CD-1	Harlan, UK	2
Fitchett et al. (2009)	CD-1	Harlan, UK	2
Hilakivi et al. (1989)	NIH Swiss	–	4 to 5
Hilakivi-Clarke and Lister (1992)	NIH Swiss	–	5
Horii et al. (2017)	C57BL/6NCrSlc	Japan SLC Inc., Shizuoka	4
Kunkel and Wang (2018)	C57BL/6	–	3 to 5^*^
Larrieu et al. (2017)	C57BL/6J	Charles River	4
Palanza et al. (2001)	Swiss CD-1^∧^	Charles River, Italy	3^*^
Pallé et al. (2019)	C57BL/6J	Envigo, UK	4
Saldívar-González et al. (2007)	BALB/cJ^∧^	–	3 and 9
Sá-Rocha et al. (2006)	C57BL/6	–	2
Varholick et al. (2018)	C57BL/6ByJ	Charles River, France	4 to 5
Varholick et al. (2019)	RjOrl:SWISS	Janvier Labs, France	3
Vekovishcheva and Sukhotina (2000)	White Outbred	Rappolovo, Russia	3
Wang et al. (2011)	C57BL/6	–	4
Zhou et al. (2017)	C57BL/6J	Shanghai laboratory animal center	4

∧ denotes mice bred in house;

* *denotes litter-mates, – denotes information not available. For the “group-size” column the conjunction “and” denotes that both group sizes were studied, where the conjunction “to” denotes that range of group sizes that were studied but not systematically varied*.

### Risk of Bias and Quality Assessment

**Figure 1:**
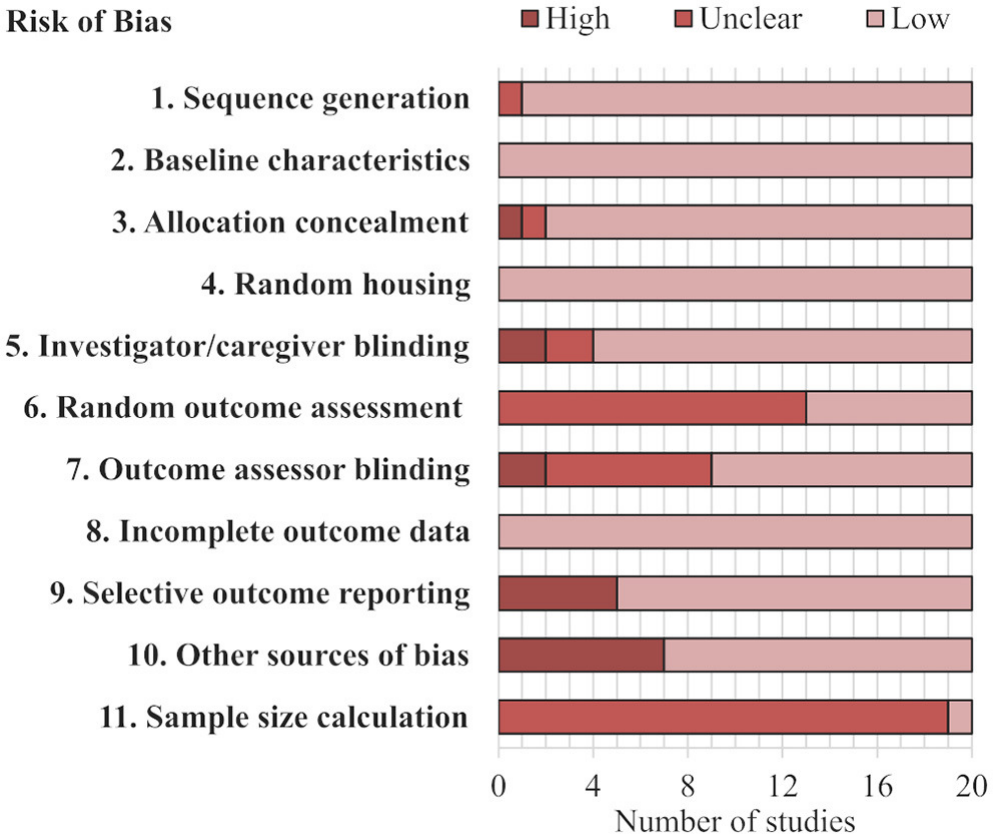
Stacked bar chart of risk of bias assessment. Each row represents a type of bias assessed for the studies. Further details can be found in Supplementary Table 1.

The risk of bias evaluation of the 20 included articles in this review is reported in Figure 1 and Supplementary Table 3. All 20 studies had a low risk of bias concerning three indices: baseline characteristics, incomplete outcome data, and random housing. A total of 19 studies had a low risk of bias for sequence generation, the remaining article had an unclear risk of bias because it did not explicitly describe the method for determining dominance. Another combination of 19 articles had low risk of bias from allocation concealment, meaning that the social dominance status of the animal was concealed during the dominance assessment method. Only one study reported a sample size calculation. The other indices had more varied distributions of low, unclear, or high risk of bias. For example, seven articles expressly stated random outcome assessment reflecting a low risk of bias, while the other 13 had unclear risk of bias. Notably, 16 and 11 of the studies had a low risk of bias from investigator blinding and outcome assessor blinding, respectively; many of the other articles had unclear risk of bias from blinding as they did not expressly describe the blinding process. An example of high risk of bias for both investigator blinding and outcome assessor blinding would be that the subordinate ranked animals always had bite-marks while dominant ranked animals had none. Importantly, seven articles had a high risk of bias from excluding cage-groups that had an unstable dominance organization or ranking, this was consistently found as “other sources of bias.” In summary, all but one study (Varholick et al., 2019) had at least one unclear (19 out of 20) and/or at least one high risk of bias (12 out of 20) (Supplementary Table 3).

### Heterogeneity in Dominance Assessment Method

The collected studies differed in their method of dominance assessment, the frequency of measuring dominance, age of animals at grouping, and the age of animals when assessing dominance—not all studies reported these metrics (Table 2). Several studies used multiple methods to determine social dominance status (Vekovishcheva and Sukhotina, 2000; Wang et al., 2011; Larrieu et al., 2017; Zhou et al., 2017).

**Table 2:** Heterogeneity in dominance assessment method for studies meeting pre-specified criteria.

**Study**	**Dominance test**	**Freq. measure dom**.	**Age at grouping**	**Age at testing**	**Time together before dom. test**
Bartolomucci et al. (2001)	Home-cage observation | Bite-marks	3 consec. days | 60 min observation	90	90	0
Bartolomucci et al. (2004)	Home-cage observation | Bite-marks	Daily | 60 min observation	100	100	0
Colas-Zelin et al. (2012)	Home-cage observation | Bite-marks	1 day | three 10 min observations	67	68	1
Ferrari et al. (1998)	Home-cage observation | Bite-marks	3 consec. Days | three 10 min observations	56	77	21
Fitchett et al. (2005a)	Home-cage observation	–	77	–	–
Fitchett et al. (2009)	Home-cage observation	14 consec. Days | 30 min observation	77	77	0
Hilakivi et al. (1989)	Presence/Absence of Bite-marks	–	–	–	–
Hilakivi-Clarke and Lister (1992)	Presence/Absence of Bite-marks	–	–	–	–
Horii et al. (2017)	Home-cage observation	4 consec. Days | 20 min after first attack	35	56	21
Kunkel and Wang (2018)	Tube-test	4 consec. Days	0	90	90
Larrieu et al. (2017)	Tube-test | Urine Marking | Home-cage observation	8 consec. days | One 20 min home-cage observation	35	70	35
Palanza et al. (2001)	Home-cage observation	> 1 consec. Day | Unclear duration	0	90	90
Pallé et al. (2019)	Tube-test	3–7 days (until stable ranks)	56	70	21
Saldívar-González et al. (2007)	Home-cage observation	5 consec. Days | 10 25–30 s	–	98	–
Sá-Rocha et al. (2006)	7 min Isolation, then Home-cage observation	3 consec. Days | 15 min observation	21	90	69
Varholick et al. (2018)	Tube-test	3 weekly tests	21	180	159
Varholick et al. (2019)	Tube-test	3 weekly tests	21	70	49
Vekovishcheva and Sukhotina (2000)	Intruder | Home-cage observation	2 days separated by 2–3 days | predetermined dominance	–	–	7
Wang et al. (2011)	Tube-test | Urine Marking | Home-cage observation | Ultrasonic Vocalization	6 consec. days | One 20 min home-cage observation	70	84	14
Zhou et al. (2017)	Tube-test | Warm-spot competition	4 consec. days	60	74	14

The most common method used in the assessment of social dominance status was home-cage behavior observation and scoring for three or more consecutive days before behavioral testing (12 out of 20) (Ferrari et al., 1998; Vekovishcheva and Sukhotina, 2000; Bartolomucci et al., 2001, 2004; Palanza et al., 2001; Fitchett et al., 2005a, 2009; Sá-Rocha et al., 2006; Saldívar-González et al., 2007; Wang et al., 2011; Horii et al., 2017; Larrieu et al., 2017). Mice that engaged in more offensive behavior (e.g., attack, chase, mount, bite) compared to defensive behavior (e.g., flee, freeze, supine posture) were rated dominant, while those that showed more defensive behavior than offensive behavior were ranked subordinate. A total of four studies of these 12 also considered bite-wounds as a sign of dominance where the subordinate incurred bite-wounds and the dominant had none (Ferrari et al., 1998; Bartolomucci et al., 2001, 2004; Colas-Zelin et al., 2012). This method of identifying dominance was used in two other studies without the provision of home-cage behavior (Hilakivi et al., 1989; Hilakivi-Clarke and Lister, 1992). Notably, one study measured home-cage behavior but only for a single day before testing general behavior (Colas-Zelin et al., 2012)—while all other home-cage behavior studies measured dominance for at least three consecutive days. The studies using home-cage observation had very inconsistent methods regarding the frequency of measuring dominance (Range = 1–14 consecutive days, Media *n* = 3 days), time spent observing dominance for each test day (5–60 min, see Table 2), the age of the animals at grouping (Range = 0–100 days of age, Media*n* = 68.5 days of age), and the time the animals were housed together before recording dominance (Range = 0–90 days, Media*n* = 10.5 days). Of the studies that evaluated home-cage behavior, three of them discarded groups where cage-mates did not have unique dominance ranks (Vekovishcheva and Sukhotina, 2000; Bartolomucci et al., 2001; Wang et al., 2011). Notably, the study by Vekovishcheva and Sukhotina (2000) experimentally formed groups with linear hierarchies by identifying the primary aggressor in a group of eight, then identifying the secondary aggressors by consecutively removing the group aggressor every 3 days until a final submissive animal that showed no aggressive behavior was left. The groups of eight were then reduced to groups of three composed of a primary aggressor, secondary aggressor, and the final submissive animal.

The next most common method for the assessment of dominance behavior involved the use of the tube-test (or competitive exclusion test) (seven out of 20) (Wang et al., 2011; Larrieu et al., 2017; Zhou et al., 2017; Kunkel and Wang, 2018; Varholick et al., 2018, 2019; Pallé et al., 2019). For this task, cage-mates are simultaneously placed on opposite ends of a long-narrow tube to impose a face-to-face conflict terminating when one cage-mate retreats backwards to their starting point. The retreating cage-mate is assigned a “loss” and its partner is assigned a “win.” These dyadic encounters are usually organized in a predetermined and random round-robin tournament. The total number of “losses” with the respective pairing compose the dominance hierarchy for each cage. Studies indicate dominance in the tube-test significantly correlates with home-cage observation (Wang et al., 2014), although some have questioned the utility of the test; namely that it doesn't always correlate with home-cage observation, animals adapt to the test over time, and it only measures a single facet of social dominance behavior (Wilson, 1968; Syme, 1974; Miczek and Barry, 1975; Benton et al., 1980; Curley, 2011; Varholick, 2019). Again, methodologies greatly varied between studies regarding the frequency of measuring dominance, the age of the animals, and the time the animals spent together before testing (see Table 2). For example, some studies measured dominance for 1 day every week for 3 weeks (Varholick et al., 2018, 2019), while others measured dominance across four or more consecutive days (Wang et al., 2011; Larrieu et al., 2017; Zhou et al., 2017; Kunkel and Wang, 2018), or every day until a group of cage-mates attained stable ranks (Pallé et al., 2019). Several studies discarded groups that had unstable hierarchies across their predetermined number of study days (Wang et al., 2011; Zhou et al., 2017).

Other methods of assessing dominance like the urine marking assay, ultrasonic vocalization, and warm-spot competition were always used in conjunction with either home-cage behavior or the tube-test but were rarely used in general [i.e., urine marking assay two out of 20 (Wang et al., 2011; Larrieu et al., 2017), ultrasonic vocalization one out of 20 (Wang et al., 2011), and warm spot competition one out of 20 (Zhou et al., 2017)]. In all cases dominance measured in these tests correlated with assessments in the home-cage or tube-test for the respective study. The urine marking assay was used for two studies (Wang et al., 2011; Larrieu et al., 2017), and involves placing two cage-mates in a novel empty cage separated by a mesh barrier with filter paper flooring. The dominant cage-mate will leave urine marks throughout their partitioned area while the subordinate will leave a pool of urine in a corner. The ultrasonic vocalization test was used once (Wang et al., 2011) in conjunction with the tube-test and home-cage observation. Here, separated males are presented with a female and 70 kHz vocalizations are recorded where the most dominant vocalizes for the longest and the subordinate often does not vocalize at all (Nyby et al., 1976). The warm-spot competition test was also used once (Zhou et al., 2017) with the tube-test, and involved presenting a group of cage-mates with a cold floored cage with a single warm-spot in the corner. Cage-mates that spent the longest time on the warm-spot were considered most dominant, with subordinate cage-mates spending the least amount of time on the warm-spot. Additional explanations of each dominance assessment method can be found in the Supplementary Text 2.

### General Composition of Behavioral Tests

The 55 individual experiments from the 20 studies were categorized into several behavioral outcome assessment domains according to Wahlsten (2011) and the studies' method sections; Exploration (*n* = 17) (Hilakivi et al., 1989; Hilakivi-Clarke and Lister, 1992; Vekovishcheva and Sukhotina, 2000; Bartolomucci et al., 2001, 2004; Palanza et al., 2001; Sá-Rocha et al., 2006; Saldívar-González et al., 2007; Wang et al., 2011; Colas-Zelin et al., 2012; Horii et al., 2017; Larrieu et al., 2017; Zhou et al., 2017; Varholick et al., 2018, 2019), Anxiety (*n* = 17) (Hilakivi et al., 1989; Hilakivi-Clarke and Lister, 1992; Ferrari et al., 1998; Vekovishcheva and Sukhotina, 2000; Bartolomucci et al., 2004; Sá-Rocha et al., 2006; Saldívar-González et al., 2007; Colas-Zelin et al., 2012; Horii et al., 2017; Larrieu et al., 2017; Varholick et al., 2018, 2019; Pallé et al., 2019), Learned Helplessness (*n* = 5) (Hilakivi et al., 1989; Hilakivi-Clarke and Lister, 1992; Saldívar-González et al., 2007; Horii et al., 2017), Cognitive (*n* = 8) (Fitchett et al., 2005a, 2009; Colas-Zelin et al., 2012; Varholick et al., 2018, 2019), Social (*n* = 2) (Zhou et al., 2017; Kunkel and Wang, 2018), and Sensory (*n* = 6) (Colas-Zelin et al., 2012; Zhou et al., 2017). Because tests measuring exploration or anxiety were most frequent, they were further sub-divided into a specific test and other category: for exploration, Open Field (*n* = 11) and Other Exploration tests (*n* = 6); and for anxiety, Elevated Plus-Maze (*n* = 13), and Other Anxiety tests (*n* = 4). Several studies met all inclusion and exclusion criteria, yet reported null effects without the provision of data: Exploration (*n* = 3/16) (Wang et al., 2011; Colas-Zelin et al., 2012; Zhou et al., 2017), Anxiety (*n* = 4/17) (Bartolomucci et al., 2004; Colas-Zelin et al., 2012) Cognitive (*n* = 4/8) (Fitchett et al., 2009; Colas-Zelin et al., 2012), Social (*n* = 1/2) (Zhou et al., 2017), Sensory (*n* = 5/6) (Colas-Zelin et al., 2012; Zhou et al., 2017). These studies are included in the tables but are excluded from the meta-analyses (Bartolomucci et al., 2004; Wang et al., 2011; Zhou et al., 2017). Only 2 studies used males and females (Varholick et al., 2018, 2019), while the remaining 18 studies exclusively studied males.

### Analysis of Exploration Behavior

Meta-analyses for exploration behavior were divided into two separate analyses for behavior in the open field and other exploration tests. The category designated as “other” in exploration behavior was not used in meta-analyses due to high heterogeneity in paradigm methodologies between studies (e.g., novel object exploration, hole-board crossings, and activity meter). For the meta-analysis that was run, open field behavior had heterogeneous metrics reported between studies. Thus, we prioritized common metrics for the data analysis. The most common metric for the open-field was total distance traveled, followed by number of crossings, then velocity; and for general exploration the number of crossings was most common followed by total distance.

**Figure 2:**
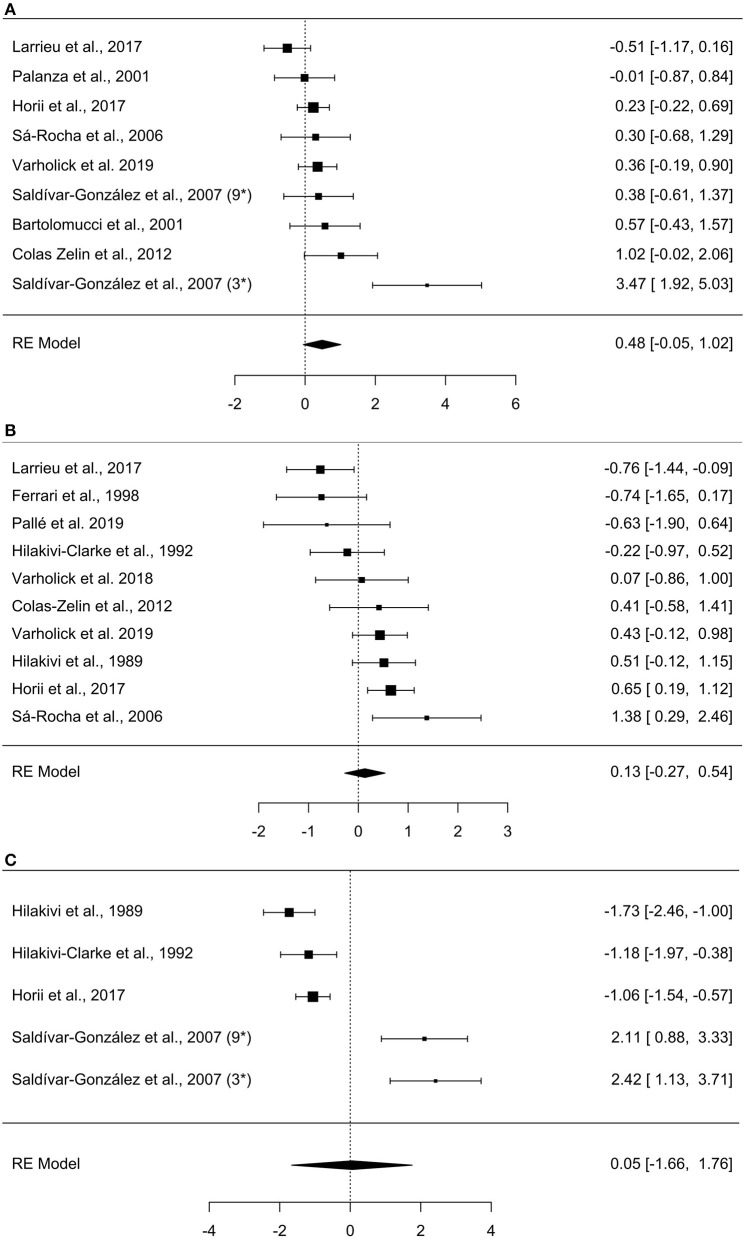
Forest plots of meta-analyses. **(A)** Open field, **(B)** Elevated plus-maze, **(C)** Porsolt forced swim test. Observed effect sizes and 95% confidence intervals are provided in the right column. Negative effect sizes represent increased values for subordinate mice (e.g., increased exploration in the open field), while positive effects represent increased values for dominants. The overall effect size is denoted by the diamond symbol. The study by Saldívar-González et al. (2007) is marked with 3^*^ and 9^*^ to denote the effects of the separate group-sizes of 3 and 9.

The meta-analysis on exploration in the open field estimated a medium effect size of 0.484 (k = 9, se = 0.273) that was not statistically significant (*p* = 0.077, 95% CI = −0.05, 1.02, Figure 2A), with experiments generally finding a small and statistically non-significant effect with dominant mice exploring more than subordinates (7/9). The other two experiments found a large statistically significant effect of dominants exploring more than subordinates (Saldívar-González et al., 2007) or a small non-significant effect in the opposite direction (Larrieu et al., 2017). There was significant between-study heterogeneity (Tau^2^ = 0.466, se = 0.332, df = 8, *p* = 0.002). For the experiments categorized as “other” within the exploration domain, the mean differences between dominant and subordinate mice, the pooled standard deviations, and Hedge's g values were calculated for each study (Table 3).

**Table 3:** Heterogeneity in effect sizes and directionality of effect for studies meeting pre-specified criteria for exploratory and anxiety domains.

**Domain**	**Study**	**Paradigm**	**Dom**	**Sub**	**Mean Diff**.	**Pooled SD**	**g**	**Dom v. Sub**
Exploration	Pallé et al., 2019	General Exploration	5	5	72.5	76.20	0.95	<
	Varholick et al., 2018	Object Exploration	10	7	298.03	1288.46	0.23	<
	Bartolomucci et al., 2004	Free Exploration	8	8	–	–	–	=
	Hilakivi et al., 1989	Hole-board	18	22	9.11	18.66	0.49	>
	Hilakivi-Clarke and Lister, 1992	Hole-board	10	23	19.44	19.50	1.0	>
	Vekovishcheva and Sukhotina, 2000	Actometer	20	20	32.10	28.78	1.12	>
Anxiety	Colas-Zelin et al., 2012	Light/Dark Box	8	8	–	–	–	=
	Saldívar-González et al., 2007 (9*)	Defensive Burying	8	8	5.44	25.77	0.22	>
	Vekovishcheva and Sukhotina, 2000	Shuttle box	20	20	6.3	11.46	0.55	>
	Saldívar-González et al., 2007 (3*)	Defensive Burying	8	8	19.24	22.59	0.85	>

The directionality in the dominance subordinance relationship is noted in the “Dom vs. Sub” column and does not necessarily reflect statistical significance. Also, studies marked with a “=” sign in the ‘Dom vs. Sub' column did not report values in their study but reported that there was no significant difference between groups. A Hedge's g value of more than 1 indicates a difference >1 standard deviation. Data ordered by direction of effect and ascending Hedge's g, similar to forest plots. The study by Saldívar-González et al. (2007) is marked with 3* and 9* to denote the effects of the separate group-sizes of 3 and 9.

### Analysis of Anxiety Behavior

As in the exploration domain, analyses in the anxiety behavior domain were sub-divided into elevated plus-maze and other anxiety tests. A meta-analysis was performed for the elevated plus-maze, while tests in the “other” category were not used in meta-analyses due to high heterogeneity in paradigm methodologies between studies (e.g., light/dark box, defensive burying, shuttlebox). The elevated plus-maze had heterogeneous metrics reported between studies, thus we prioritized common metrics; percent entries in open arms was most common, followed by percent duration in open arms, and finally number of open arm entries. Notably, not all studies directly reported “percent” but provided enough information to calculate the percentage (e.g., number of open arm entries divided by total entries), allowing more studies to have more comparable metrics. The meta-analysis on elevated plus-maze behavior yielded a small effect size of 0.132 (k = 10, se = 0.206) that was not statistically significant (*p* = 0.522, 95% CI= −0.27, 0.54, Figure 2B). With about an equal number of experiments finding a small and non-significant effect in contradicting directions (4 indicating increased anxiety for dominants and three increased anxiety for subordinates), another two with significant effects in contradicting directions (Horii et al., 2017; Larrieu et al., 2017), and one study finding virtually no effect (Varholick et al., 2018). There was, again, significant between-study heterogeneity (Tau^2^ = 0.256, se = 0.197, df = 9, *p* = 0.003). For the experiments categorized as “other” within the anxiety domain, the mean differences between dominant and subordinate mice, the pooled standard deviations, and Hedge's g values were calculated for each study (Table 3).

### Analysis of Learned Helplessness Behavior

The only tests that measured learned helplessness in this review were those using the Porsolt forced swim test. Five studies conducted this test in relation to dominance and thus satisfied our criteria for inclusion. All studies reported the same metric, duration immobile, thus the meta-analysis was limited to comparing duration immobile between studies. The meta-analysis on the Porsolt forced swim test estimated a small effect size of 0.0480 (k = 5, se = 0.872) that was not statistically significant (*p* = 0.956, 95% CI = −1.66, 1.76, Figure 2C). This was attributable to contradictory statistically significant findings across experiments with three reporting subordinates spend more time immobile (Hilakivi et al., 1989; Hilakivi-Clarke and Lister, 1992; Horii et al., 2017) and two reporting dominants spend more time immobile—albeit the latter experiments were from the same study (Saldívar-González et al., 2007). There was again substantial between-study heterogeneity (Tau^2^ = 3.574, se = 2.691, *p* = 0.001).

A secondary set of forest-plots for open field, elevated plus-maze, and Porsolt forced swim test focusing on relevant study characteristics (i.e., strain, group-size, and dominance assessment method) can be found in the supplement (Supplementary Figure 2).

### Analysis of Cognitive, Social, and Sensory Behavior

Given a high degree of heterogeneity across behavioral outcome assessments and inconsistent reporting between studies within the separate domains; cognitive, social, and sensory, the data were insufficient for meta-analyses. However, for comparison to the previous meta-analyses and further discussion, the mean differences, pooled standard variations, and Hedge's g values are reported in Table 4. Experiments marked with a “=” sign in the “Dom vs. Sub” column did not report values in their study, they just reported that there was no significant difference between groups.

**Table 4:** Heterogeneity in effect sizes and directionality of effect for studies meeting pre-specified criteria for cognitive, social, and sensory domains.

**Domain**	**Study**	**Paradigm**	**Dom**	**Sub**	**Mean Diff**.	**Pooled SD**	**g**	**Dom v. Sub**
Cognitive	Varholick et al., 2019	Novel object discrimination	26	26	0.11	0.12	0.88	<
	Varholick et al., 2018	Novel object discrimination	10	7	0.02	0.21	0.07	<
	Fitchett et al., 2009	T-Maze	9	9	–	–	–	=
	Colas-Zelin et al., 2012	Fear conditioning	8	8	–	–	–	=
	Colas-Zelin et al., 2012	Morris water maze	8	8	–	–	–	=
	Colas-Zelin et al., 2012	Lashley III Maze	8	8	–	–	–	=
	Colas-Zelin et al., 2012	Odor Discrimination	8	8	–	–	–	=
	Fitchett et al., 2005a	T-Maze	6	6	0.41	0.63	0.65	>
Social	Zhou et al., 2017	Social memory	–	–	–	–	–	=
	Kunkel and Wang, 2018	Social memory	8	8	15.14	11.38	1.39	>
Sensory	Vekovishcheva and Sukhotina, 2000	Hot-plate	6	6	0	1.86	0	=
	Colas-Zelin et al., 2012	Hot-plate	8	8	–	–	–	=
	Colas-Zelin et al., 2012	Balance beam	8	8	–	–	–	=
	Colas-Zelin et al., 2012	Grip strength	8	8	–	–	–	=
	Zhou et al., 2017	Grip strength	–	–	–	–	–	=
	Colas-Zelin et al., 2012	Balance pole	8	8	–	–	–	=

Data ordered by direction of effect and ascending Hedge's g, similar to forest plots. The directionality in the dominance subordinance relationship is noted in the “Dom vs. Sub” column and does not necessarily reflect statistical significance. Also, studies marked with a “=” sign in the “Dom vs. Sub” column did not report values in their study but reported that there was no significant difference between groups. A Hedge's g-value of more than 1 indicates a difference >1 standard deviation.

Similar to the meta-analyses for exploration and anxiety behavior, experiments generally reported no large effects between dominant and subordinate mice for cognitive, social, or sensory behavior. Regarding cognitive behavior, most experiments reported small effect sizes with no statistical significance (*n* = 6/8), albeit five of those experiments did not report the data (Fitchett et al., 2009; Colas-Zelin et al., 2012). Only two studies measured social behavior, beyond dominance, with opposing results; one with no effect and no reported data (Zhou et al., 2017) and another with a large effect indicating dominant mice had increased social memory compared to subordinate mice (Kunkel and Wang, 2018). Finally, regarding sensory behavior all experiments found virtually no effect between dominant mice and subordinate mice, with 5 out of 6 not reporting the data.

## Discussion

This systematic review and meta-analysis revealed limited evidence to support the notion that a clear difference exists between dominant and subordinate male laboratory mice on standard measures of behavior commonly used in biomedical research. The 55 experiments from 20 published papers used to inform this review were heterogeneous concerning strain, group-size, age of testing dominance, and their methods for assessing dominance. Such heterogeneity likely increased the generalizability of our assessment, but the unsystematic nature of this heterogeneity may have also clouded our understanding on which genetic, environmental, and developmental factors might be most important when considering dominance and behavior. Most studies (12 out of 20) had at least one high risk of bias, and only a single study (Varholick et al., 2019) had neither high nor unclear risks of bias. A number of studies failed to report experimental data and/or exclusively studied groups of mice with stable dominance hierarchies thereby excluding other dominance organizations (e.g., despotic or unclear). Studies were also quite heterogenous regarding the domains of behavior measured; exploration, anxiety, learned helplessness, cognitive, social, and sensory behavior domains. With the domains of exploration, anxiety, and learned helplessness being the most frequent outcomes, we were able to conduct meta-analyses finding that dominant and subordinate mice tend to have small to medium effect size differences in exploratory behavior in the open field and elevated plus maze, but none of the summary effect sizes reached statistical significance. Systematic review and meta-analyses concerning the Porsolt forced swim test (i.e., learned helplessness) found extremely large and paradoxical patterns of differences between dominant and subordinate mice across studies, which overall led to a non-significant summary effect size. Comparison of Hedge's g values for the other behavioral domains which were too heterogeneous to consider in meta-analyses yielded a similar pattern of results found in exploration, anxiety, and learned helplessness behavior.

Our overall assessment of risks of bias highlighted potential issues which precludes us from drawing firm conclusions about the relationships between social dominance status and common measures of behavior. Five domains of bias considered in this review were (i) selection bias, (ii) performance bias, (iii) detection bias, (iv) reporting bias, and (v) other bias (Hooijmans et al., 2014). Risk from (i) selection bias was generally low and was assessed by baseline characteristics, sequence generation, and allocation concealment. All studies had low risk of bias from baseline characteristics since animals were randomly distributed across housing and then dominance was assessed. However, one study (Fitchett et al., 2005a) did not explicitly specify how dominance was determined, making the assessment of selection bias unclear for sequence generation and allocation concealment. Specifically, the study was a brief report that failed to describe any methods but cited a publication (Fitchett et al., 2005b) when referring to “further details” of their urinary corticosterone assay. The cited publication also measured social dominance, but whether the included study and the cited publication used the exact dominance method was unclear. Another study determined dominance on the first day of assessment and then confirmed dominance each subsequent day (Bartolomucci et al., 2004), which increased the risk of bias for allocation concealment. Risk from (ii) performance bias was also generally low and was assessed by random housing and blinding of dominance rank to housing and husbandry staff. All studies randomly allocated mice to cages upon arrival to their lab or the start of the experiment. Notably, one study (Vekovishcheva and Sukhotina, 2000) randomly housed mice in groups of 8, assessed dominance rank, and then reduced groups to 3 composed of a dominant, sub-dominant, and subordinate. This could be considered a risk of bias from non-random housing but was categorized as “other bias” since animals were randomly housed prior to dominance assessment. Several instances of high risks of bias from determining dominance solely by bite-marks (Hilakivi et al., 1989; Hilakivi-Clarke and Lister, 1992) were concerning since all individuals handling the mice would immediately recognize whether they were dominant or subordinate and might handle them differently. This source of bias may be unavoidable due to the nature of social dominance, however, including other methods of dominance assessment beyond bite-wounds could reduce the risk. Risk from (iii) detection bias was mostly unclear throughout studies with several instances of high risk of bias, and was assessed by randomization of outcome assessment, and investigator blinding during outcome assessment. Most studies (13 out of 20) (Hilakivi-Clarke and Lister, 1992; Ferrari et al., 1998; Vekovishcheva and Sukhotina, 2000; Palanza et al., 2001; Saldívar-González et al., 2007; Fitchett et al., 2009; Wang et al., 2011; Colas-Zelin et al., 2012; Horii et al., 2017; Larrieu et al., 2017; Zhou et al., 2017; Kunkel and Wang, 2018; Pallé et al., 2019) did not report whether dominance rank was counterbalanced or considered in the order of testing, making randomization of outcome assessment unclear. More than a third of studies (seven out of 20) (Vekovishcheva and Sukhotina, 2000; Bartolomucci et al., 2004; Fitchett et al., 2005a, 2009; Saldívar-González et al., 2007; Zhou et al., 2017; Kunkel and Wang, 2018) also did not explicitly state whether the investigator assessing outcome measures was blinded to the dominance rank of the animals tested (if this was possible). Risk from (iv) reporting bias occurred in a quarter of the studies (five out of 20) (Bartolomucci et al., 2004; Fitchett et al., 2009; Wang et al., 2011; Colas-Zelin et al., 2012; Zhou et al., 2017), which selectively reported statistically significant differences and just reported “no statistical difference” for non-significant effects without the provision of data. This prevented us from determining the effect size for these outcome measures. One (v) “other bias” that was common across studies was discarding social groups that did not form clear dominance hierarchies (seven out of 20 studies) (Vekovishcheva and Sukhotina, 2000; Bartolomucci et al., 2001; Palanza et al., 2001; Sá-Rocha et al., 2006; Wang et al., 2011; Horii et al., 2017; Zhou et al., 2017). Several studies have estimated that male laboratory mice form stable groups with unique ranks 60% of the time, while unstable or despotic structures are formed in the other 40% (Wang et al., 2011, 2014; Varholick et al., 2019). Thus, discarding unstable or despotic structures would bias our understanding of the general relationship between social dominance and behavioral tests. Finally, only one study reported conducting a sample size calculation (Varholick et al., 2019). This is concerning because it is unclear whether studies considered effect sizes before designing and conducting the experiment (Carneiro et al., 2018). Moreover, the study that did conduct the sample size calculation (Varholick et al., 2019) considered the likely distribution of stable (estimated at 60%) and unstable groups (estimated at 40%) of social dominance (Wang et al., 2011; Colas-Zelin et al., 2012; Varholick et al., 2018). This method decreases the risk of bias from the stability of dominance, something few studies considered throughout this review.

The general finding of non-significant and relatively small differences is contrary to the theoretical and individual experimental findings that social status shapes behavior and physiology across life-histories in male laboratory mice (Williamson et al., 2016a, 2017a,b; Lee et al., 2018, 2019b; Williamson et al., 2019), and the large effects of the social environment reported in other species (Snyder-Mackler et al., 2020). Over the years, researchers have reported many large effects associated with dominance in male laboratory mice for measures of the hypothalamic-pituitary-adrenal (HPA) and hypothalamic-pituitary-gonadotropic (HPG) axes (Louch and Higginbotham, 1967; Bronson, 1973; Ely and Henry, 1978; Williamson et al., 2017a,b), the mesolimbic dopaminergic pathway (Balog et al., 2014; Larrieu et al., 2017; Papilloud et al., 2020), and exploratory behavior (Sloan Wilson et al., 1994). Some of these studies have used a more ethological approach when studying social behavior with large group sizes (>10) in complex housing with additional structures and space (Williamson et al., 2016b)—albeit larger groups and complex housing may not generalize to common laboratory conditions or experimental designs in biomedical science. To better understand these effects, individual experiments could consider how larger group sizes or complex housing are related to dominance in comparison to more standard laboratory conditions.

It is likely that the discrepancy between the findings of this systematic review and the aforementioned literature is partially due to study heterogeneity and the sensitivity of dominance structures to differences in genetics and the environment. That is, some combinations are more likely to have more divergent social dominance statuses with consequent effects than others. Studies included in the meta-analyses of this review greatly varied in the reported factors of strain, group-size, age, housing and husbandry conditions, and even method of assessing dominance (see Supplementary Text 2 for a brief description of each method from this systematic review). They also greatly varied in reported effect sizes ranging from small to extremely large effects. These inconsistencies highlight the potential sensitivity of dominance to other variables across a “reaction norm” and development (Woltereck, 1909; Wahlsten, 2010; Voelkl and Würbel, 2016; Voelkl et al., 2020). Indeed, previous studies considering multiple strains (Mondragón et al., 1987), group-sizes (Schuhr, 1987; Saldívar-González et al., 2007; Williamson et al., 2017a), and ages (Bartolomucci et al., 2004) have all shown robust interactions with dominance relationships—albeit not directly for the metrics reviewed in the current study. This is surprising, however, it is possible that different dominance assessment methods highlight specific facets of social dominance more than others, thereby increasing the chance of finding seemingly congruent effects (Bernstein, 1981; Varholick et al., 2019).

Apart from differences in experimental design and assessment, between-study variation might be due to tests measuring facets of anxiety that are sensitive to common laboratory environmental variables like the familiarity of the experimenter, position of the home-cage on the rack, or arousal state of the animal immediately before the test (Izídio et al., 2005). No studies in this review explicitly reported controlling for these variables. Some researchers have suggested that differences between studies can be, in part, evaluated by authorship heterogeneity since related co-authors can publish multiple papers with similar methods and effect sizes (Moulin and Amaral, 2020). However, this rarely occurred in the current review. Besides the same author publishing two studies with the Porsolt forced swim test (Hilakivi et al., 1989; Hilakivi-Clarke and Lister, 1992), there was one occurrence in the open field analysis across 8 studies (Bartolomucci et al., 2001; Palanza et al., 2001) and two for the elevated-plus maze across 10 studies (Hilakivi et al., 1989; Hilakivi-Clarke and Lister, 1992; Varholick et al., 2018, 2019). Given the low occurrence of authorship relatedness, that most studies reported no significant differences, and study characteristics greatly differed; we posit that authorship relatedness cannot accurately capture a lab effect and future empirical research is necessary.

Given this perspective, a logical next step to disentangle idiosyncratic results would be to replicate the experiments involving the elevated plus-maze and Porsolt forced swim test—where individual studies reported large effect sizes—while also considering different strains, group-sizes, ages, and dominance methodologies as heterogenization factors to further explore these effects, followed up by appropriately powered experiments for hypothesis testing (Voelkl et al., 2020). Consideration for other dominance organizations (e.g., despotic, double-dominant, double-subordinate, or open) may also be helpful as such evaluations were explicitly performed in two of the included studies (Horii et al., 2017; Varholick et al., 2019). This will allow these other variables to be considered against the backdrop of reproducibility while providing mechanistic insight into which combinations of genetics, environments, and developmental phases may be relevant to dominance relationships and thus, require further investigation. Such an understanding may also provide a platform for increasing the chance of finding an effect if one exists concerning the domains of behavior evaluated here—cognitive, social, and sensory—but where too few studies and inconsistency in the pattern of results of behavior precluded firm conclusions. Moreover, these studies could be beneficial in the formulation of studies considering dominance relationships in female laboratory mice—which this review found to be critically lacking.

## Data Availability Statement

The datasets extracted from publications and used for the meta-analyses for this study can be found in the figshare at: doi: 10.6084/m9.figshare.13313258.

## Author Contributions

This research was conceptualized by JV, JB, BV, and HW. Methodology by JV, JB, BV, and AJ. Investigation by JV and AJ. Risk of bias sections by AJ and JV. Formal analysis and original draft by JV. Reviewing and editing by all authors.

## Conflict of Interest

The authors declare that the research was conducted in the absence of any commercial or financial relationships that could be construed as a potential conflict of interest.
