# Supplementary material for: A Systematic Review and Meta-Analysis of the Relationship Between Social Dominance Status and Common Behavioral Phenotypes in Male Laboratory Mice
Source: Front Behav Neurosci. 2021 Jan 20;14:624036. doi: 10.3389/fnbeh.2020.624036 (PMC7855301; doi:10.3389/fnbeh.2020.624036)
Supplement: Supplementary file 1 [file Data_Sheet_1.DOCX]

Supplementary Material

# Supplementary Data

1_FinalData.xlsx : All data collected for meta-analyses by the two investigators (JV and AJ)
2_MetaAnalysisMergedData.omv : Jamovi file for meta-analyses
3_BiasSheet.xlsx : All bias assessments by the two investigators (JV and AJ)
4_All Extracted Data.xlsx : All data extracted for meta-analyses, tables, etc. (AJ and JV)

# SI Text 1. Study Protocol

## Search Procedure

First, we determined which keywords in our search resulted in relevant publications for the review. To do so, we conducted multiple searches in the title and abstract fields of the databases; PubMed, Web of Science, and EMBASE (see sub-section ‘Other Searches’). We then screened the results for the presence of a predetermined list of papers that we felt should be included in the review. This process served strictly to devise the search strategy and remove as many irrelevant publications as possible without compromising the publications relevant to the review. Indeed, “dominance” or “dominant” are commonly used words throughout science regardless of their association with social dominance status (e.g. dominant allele, dominant eye, etc.). Below is a list of the predetermined publications for this screening process:

Hilakivi-Clarke, L. A. & Lister, R. G. Are there preexisting behavioral characteristics that predict the dominant status of male NIH mice (Mus musculus)? J. Comp. Psychol. 106, 184–189 (1992).

D’Amato, F. R. Effects of male social status on reproductive success and on behavior in mice (Mus musculus). J. Comp. Psychol. 102, 146–51 (1988).

Wang, F. et al. Bidirectional Control of Social Hierarchy by Synaptic Efficacy in Medial Prefrontal Cortex. Science (80-. ). 334, 693 (2011).

Hilakivi, L. A. et al. Behavioral, hormonal and neurochemical characteristics of aggressive α-mice. Brain Res. 502, 158–166 (1989).

Kunkel, T. & Wang, H. Socially dominant mice in C57BL6 background show increased social motivation. Behav. Brain Res. 336, 173–176 (2017).

Larrieu, T. et al. Hierarchical Status Predicts Behavioral Vulnerability and Nucleus Accumbens Metabolic Profile Following Chronic Social Defeat Stress. Curr. Biol. 2202–2210 (2017). doi:10.1016/j.cub.2017.06.027

Horii, Y. et al. Hierarchy in the home cage affects behaviour and gene expression in group-housed C57BL/6 male mice. doi:10.1038/s41598-017-07233-5

Howerton, C. L., Garner, J. P. & Mench, J. A. Effects of a running wheel-igloo enrichment on aggression, hierarchy linearity, and stereotypy in group-housed male CD-1 (ICR) mice. Appl. Anim. Behav. Sci. 115, 90–103 (2008).

Colas-Zelin, D. et al. The imposition of, but not the propensity for, social subordination impairs exploratory behaviors and general cognitive abilities. Behav. Brain Res. 232, 294–305 (2012).

This trial/error and adjust method, lead to the following search strategy:

(anxiety OR arousal OR learned helplessness OR explor* OR choice OR learn* OR cognition OR preference OR motor OR pain OR maze) AND ("social status" OR "social rank" OR "social dominance" OR "dominance hierarchy" OR "social hierarchy" OR submiss*) AND (mouse OR mus OR mice)

From a search conducted on September 20th 2019, PubMed found 360; Web of Science found 303; and Embase found 407 papers matching the search criteria. After merging all articles in a single database on Mendeley, 374 papers were determined to be triplicate or duplicate, thus there were 696 unique papers in total to be screened for relevance.

## Screening for potentially relevant articles

The titles and abstracts of 30 randomly selected papers from the 696 (~4%) were read to determine criteria necessary to determine relevance. Thus, we explored ~4% of the studies and further refined our criteria using these papers. From this exploration, the following exclusion criteria were determined:

Exclusion criteria for relevance:

- Did not study mice (Mus musculus, Mus domesticus)
- Mice isolation housed
- Chronic/repeated social defeat done experimentally (e.g. once a day for 20 days)
- No measurement of same-sex social interactions (some mice bred for social dominance and compared, however, these studies do include wild-type controls)
- Part of symposium/conference, or review
- Not English

*If it was not clear whether a study met this removal criteria, then it was included as potentially relevant.

Following this first phase of exclusion criteria, for relevancy, 152 publications were determined as potentially relevant. All 152 of these papers’ methods sections were read through and the methods of dominance were reviewed and included in SI Text 2.

## Inclusion/Exclusion Criteria for Screening

The methods sections of 8 randomly selected papers from the 152 (~5%) were screened to determine necessary inclusion/exclusion criteria. Again, this was an exploratory method to ensure that we were capturing all possible studies to answer our research question. The inclusion and exclusion criteria are listed below. Notice, this part of the screening process was done in two phases to assure that our criteria were not too strict or too relaxed.

Inclusion:

- Were mice housed in groups/pairs for 2 weeks or more?
- Did they measure social dominance using the tube-test or fighting/chasing in the home-cage?
- Do they measure behavioral phenotypes of the following aspects; anxiety, arousal, learned helplessness, exploration, preference, learning/cognition, motor, pain, social?

Exclusion

- Dominance relationships measured between non-cagemates
- Were any treatments administered in addition to the inclusion criteria above? If yes, then only data from the control group is used.
- Data from same study published more than once?

# Figure 1. Flow Diagram

# SI Table 1

**SYRCLE Risk of Bias Tool (modified for current study)**

| **Item** | **Type of bias** | **Domain** | **Reviewer question** | **Example of bias (answer of no to reviewer question)** |
| --- | --- | --- | --- | --- |
| **1** | Selection Bias | Sequence generation | Was the allocation of dominant/subordinate categories adequately generated and applied? | *Method of dominance was a secondary citation and unclear* |
| **2** | Selection Bias | Baseline characteristics | Were the groups similar at baseline or were they adjusted for confounders in the analysis? | *No bias in studies* |
| **3** | Selection Bias | Allocation concealment | Was the allocation of dominant and subordinate adequately concealed? | *Dominance determined on first day and confirmed each subsequent day.* |
| **4** | Performance Bias | Random Housing | Were the animals randomly housed during the experiment? | *No bias in studies^#^* (but see item 10) |
| **5** | Performance Bias | Blinding | Were the investigators or caregivers blinded from who was dominant or subordinate during the experiment? (e.g. ID Cards) | *Subordinate ranked animals always had bite-wounds while dominants had none.* |
| **6** | Detection Bias | Random outcome assessment | Were animals selected at random for outcome assessment? | *No bias in studies, this was often unclear because it was unreported* |
| **7** | Detection Bias | Blinding | Was the outcome assessor blinded? | *Subordinate ranked animals always had bite-wounds while dominants had none.* |
| **8** | Attrition Bias | Incomplete outcome data | Were incomplete data adequately addressed? | *No bias in studies.* |
| **9** | Reporting Bias | Selective outcome reporting? | Are reports of the study free of selective outcome reporting? | *There was no effect of exploration in open field (n.s. unreported).* |
| **10** | Other Bias | Other sources of bias? | Was the study apparently free of other problems that could result in high risk of bias? | *Animals with unclear dominance or subordinance rank were excluded*. |
| **11** | Quality | Sample size calculation | Did the study conduct a sample size calculation? | *No bias in studies, was often unclear because it was unreported.* |

# SI Table 2. Summary table (part 1 of 2)

# SI Table 2. Summary table (part 2 of 2)

# SI Table 3. Summed scores for Risk of Bias for each study

| **Study** | **Low Risk** | **Unclear Risk** | **High Risk** |
| --- | --- | --- | --- |
| Bartolomucci et al., 2001 | 9 | 1 | 1 |
| Bartolomucci et al., 2004 | 6 | 3 | 2 |
| Colas-Zelin et al., 2012 | 8 | 2 | 1 |
| Ferrari et al., 1998 | 9 | 2 | 0 |
| Fitchett et al. 2005 | 6 | 5 | 0 |
| Fitchett et al. 2009 | 7 | 3 | 1 |
| Hilakivi et al., 1989 | 8 | 1 | 2 |
| Hilakivi-Clarke et al., 1992 | 7 | 2 | 2 |
| Horii et al., 2017 | 8 | 2 | 1 |
| Kunkel & Wang 2018 | 8 | 3 | 0 |
| Larrieu et al., 2017 | 9 | 2 | 0 |
| Palanza et al., 2001 | 8 | 2 | 1 |
| Pallé et al., 2019 | 9 | 2 | 0 |
| Saldivar-Gonzalez et al., 2007 | 8 | 3 | 0 |
| Sá-Rocha et al., 2006 | 9 | 1 | 1 |
| Varholick et al., 2018 | 10 | 1 | 0 |
| Varholick et al., 2019 | 11 | 0 | 0 |
| Vekovischeva et al., 2000 | 7 | 3 | 1 |
| Wang et al. 2011 | 7 | 2 | 2 |
| Zhou et al., 2017 | 6 | 3 | 2 |

#
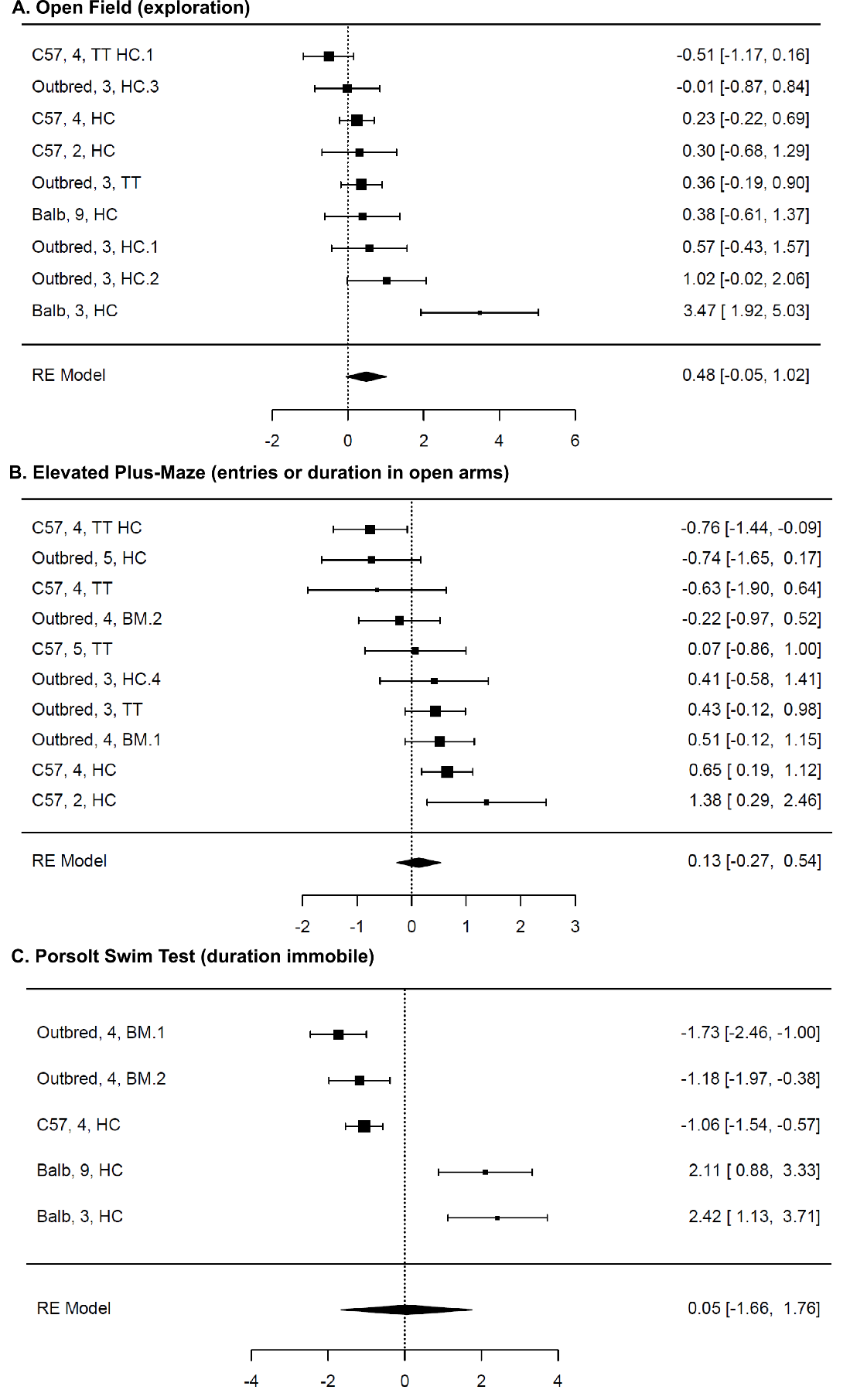
SI Figure 2. Forest plot with secondary variables

Outbred, C57, or Balb denotes strain; number denotes group-size, TT denotes tube-test, HC denotes home-cage observation. BM denotes bite marks, “.1” notation denotes different studies with identical variable

# SI Text 2. Dominance assessment methods

Home-cage observation:
Home-cage observation is considered the gold-standard for assigning dominant and subordinate mice. This is often done from video to ensure inter- and intra-rater reliability, but can be accomplished live with multiple coders (Williamson et al., 2016). With both methods, cage-mates are typically individually marked on the fur or tail and behaviors like chasing and biting are assigned as offensive, while supine or upright freeze postures, fleeing, and squeaking are assigned as defensive behaviors. The animal with the most frequent defensive behaviors is often assigned subordinate and the partner is given the dominant assignment. Due to the unpredictable and burstiness nature of agonistic behaviors composing the dominance assignments, continuous or scanning methods are used to code the time-period rather than interval coding. In almost all cases, social interactions are observed across several days with either dominance assignment occurring after all collected observations or assignment from the first day of observation and confirmation of assignment for all subsequent observations.
Notably, since the publication studying mouse agonistic behavior with high-speed videography (Banks, 1962), many believe common cameras cannot properly identify when a bite occurs or distinguish a counter-attack from an initial attack. Thanks to modern technology this is not the case. The original publication recorded video at 62 frames per second (fps), and today many digital cameras record 60fps as regular speed while 90fps and higher is today’s current “high-speed”.

Tube-test:
Published protocols and explanations of this test are widely available and should be replicated to guarantee best results (Fan et al., 2019; Varholick, 2019). To perform the task, cage-mates are simultaneously placed on opposite ends of a long-narrow tube to impose a face-to-face conflict terminating with one cage-mate retreating backwards to their starting point. This cage-mate is assigned a ‘loss’ and trials continue with the same pair and additional cage-mate pairings, alternating sides, until all cage-mates are tested. The total number of ‘losses’ with the respective pairings compose the dominance hierarchy for the cage. Extensive habituation of the method is necessary to remove the confound of experimenter handling. Some have questioned the utility of the tube-test noting it can be confounded by repeated testing (Benton et al., 1980; Miczek & Barry, 1975; Syme, 1974; Varholick, 2019; Wilson, 1968), but others have shown that rankings significantly correlate with the home-cage observations and other dominance tests (Wang et al., 2014). Indeed, dominance rankings in any scenario are an outcome of repeated social interactions.

Urine marking assay:
Mice commonly leave urine markings throughout their environments. When two cage-mates are placed into a novel cage with a mesh barrier separating them, one cage-mate typically marks the entire floor while the other only voids urine in a few pools in the cage corner (Desjardins et al., 1973). This can be seen by placing filter paper on the floor of the novel cage prior to placing the mice in the respective compartments and then using UV light to image the markings once the animals are removed. This assay can also be categorized as a special case of resource competition (see below), where space is the resource in this case.

Resource competition assays:
Dominance behavior is typically paired with competition for resources, and thus many have developed tests of food, mate, or warm-spot competition to measure dominance. Importantly, for competition to occur, the resource must be accessible by only one cage-mate at a time and there must be some deprivation of the resource to increase the probability of competition once the resource is presented. The cage-mate that spends the most time with the resource is assigned the dominant animal. For food competition, cage-mates may be presented with highly palatable food-like sweetened milk, chocolate, or a type of cookie (Malatynska & Knapp, 2005; Merlot et al., 2004). Mate competition involves presenting a female to males and measuring 70kHz ultrasonic vocalizations, where the incipient dominant animal vocalizes the longest – often the subordinate does not vocalize at all (Nyby et al., 1976). These types of tests have also been incorporated into the Noldus Intellicage, where mice compete for access to water (Benner et al., 2014).

*Agonistic behavior assay:
This test is a special case of home-cage observation where the experimenters capitalize on the increased probability of agonistic behavior immediately following bedding and cage changing (Cohn et al., 2012).

*Sequential removal of aggressor:
This test is another special case of home-cage observation where the experimenters remove the primary aggressor or cage-mate showing the most aggressive behavior, and then observe and record which cage-mate shows the most aggression in the absence of the primary aggressor (Vekovishcheva & Sukhotina, 2000). Subsequent removals determine the hierarchy.

*marks method recorded in screening process but not one of the 20 studies included in formal review

References for SI Text 2

Banks, E. M. (1962). A time and motion study of pre-fighting behavior in mice. *Journal of Genetic Psychology*, *101*(January 2015), 165–183. https://doi.org/10.1080/00221325.1962.10533622

Benner, S., Endo, T., Endo, N., Kakeyama, M., & Tohyama, C. (2014). Early deprivation induces competitive subordinance in C57BL/6 male mice. *Physiology & Behavior*, *137*, 42–52. https://doi.org/10.1016/j.physbeh.2014.06.018

Benton, D., Dalrymple-Alford, J. C., & Brain, P. F. (1980). Comparisons of measures of dominance in the laboratory mouse. *Animal Behaviour*, *28*(4), 1274–1279. https://doi.org/10.1016/S0003-3472(80)80115-1

Cohn, D. W. H., Gabanyi, I., Kinoshita, D., de Sa-Rocha, L. C., Hamada Cohn, D. W., Gabanyi, I., Kinoshita, D., & de Sa-Rocha, L. C. (2012). Lipopolysaccharide administration in the dominant mouse destabilizes social hierarchy. *Behavioural Processes*, *91*(1), 54–60. https://doi.org/10.1016/j.beproc.2012.05.008

Desjardins, C., Maruniak, J. A., & Bronson, F. H. (1973). Social rank in house mice: Differentiation revealed by ultraviolet visualization of urinary marking patterns. *Science (New York, N.Y.)*, *182*(4115), 939–941.

Fan, Z., Zhu, H., Zhou, T., Wang, S., Wu, Y., & Hu, H. (2019). Using the tube test to measure social hierarchy in mice. *Nature Protocols*, *14*, 819–831.

Malatynska, E., & Knapp, R. J. (2005). Dominant-submissive behavior as models of mania and depression. *Neuroscience and Biobehavioral Reviews*, *29*(4–5), 715–737. https://doi.org/10.1016/j.neubiorev.2005.03.014

Merlot, E., Moze, E., Bartolomucci, A., Dantzer, R., & Neveu, P. J. (2004). The rank assessed in a food competition test influences subsequent reactivity to immune and social challenges in mice. *Brain, Behavior, and Immunity*, *18*(5), 468–475. https://doi.org/10.1016/j.bbi.2003.11.007

Miczek, K. A., & Barry, H. (1975). What does the tube test measure? *Behavioral Biology*, *13*(4), 537–539.

Nyby, J., Dizinno, G. A., & Whitney, G. (1976). Social status and ultrasonic vocalizations of male mice. *Behavioral Biology*, *18*(2), 285–289.

Syme, G. J. (1974). The approach response and performance in the dominance tube. *Australian Journal of Psychology*, *26*(1), 31–36. https://doi.org/10.1080/00049537408254633

Varholick, J. A. (2019). Competitive exclusion. In J. Vonk & T. Shackelford (Eds.), *Encyclopedia of Animal Cognition and Behavior*. Springer International Publishing.

Vekovishcheva, O. Y., & Sukhotina, I. A. (2000). Co-housing in a stable hierarchical group is not aversive for dominant and subordinate individuals. *Neuroscience and Behavioral Physiology*, *30*(2), 195–200.

Wang, F., Kessels, H. W., & Hu, H. (2014). The mouse that roared: Neural mechanisms of social hierarchy. *Trends in Neurosciences*, *37*(11), 674–682. https://doi.org/10.1016/j.tins.2014.07.005

Williamson, C. M., Lee, W., & Curley, J. P. (2016). Temporal dynamics of social hierarchy formation and maintenance in male mice. *Animal Behaviour*, *115*, 259–272. https://doi.org/10.1016/j.anbehav.2016.03.004

Wilson, W. J. (1968). Adaptation to the dominance tube. *Psychonomic Science*, *10*(3), 119–120. https://doi.org/10.3758/BF03331437
